# Identification of multi-omics molecular programs from the peripheral circulation of patients with PAH

**DOI:** 10.1016/j.isci.2026.117937

**Published:** 2026-11-02

**Authors:** Vitaly O. Kheyfets, Andrew J. Sweatt, Hui Zhang, Min Li, Cheng-Jun Hu, Travis Nemkov, Maya A. Aizin, Paul Heerdt, Monika Dzieciatkowska, Daniel Stephenson, Ian S. LaCroix, Angelo D’Alessandro, Francesca I. Cendali, William M. Oldham, Kirk C. Hansen, Roham T. Zamanian, Kurt R. Stenmark

**Affiliations:** 1 Pediatric Critical Care Medicine, Developmental Lung Biology and CVP Research Laboratories, School of Medicine, University of Colorado, Aurora, CO, USA; 2 Division of Pulmonary and Critical Care Medicine, Stanford University, Stanford, CA, USA; 3 Vera Moulton Wall Center for Pulmonary Vascular Disease, Stanford University, Stanford, CA, USA; 4 Department of Craniofacial Biology, School of Dental Medicine, University of Colorado Anschutz Medical Campus, Aurora, CO, USA; 5 Department of Biochemistry and Molecular Genetics, University of Colorado Denver | Anschutz Medical Campus, Aurora, CO, USA; 6 Department of Anesthesiology, Applied Hemodynamics, Yale School of Medicine, New Haven, CT, USA; 7 Department of Medicine, Brigham and Women’s Hospital and Harvard Medical School, Boston, MA, USA

**Keywords:** pulmonary arterial hypertension, proteomics, metabolism, biomarkers

## Abstract

Pulmonary arterial hypertension (PAH) involves progressive remodeling of the pulmonary vasculature and right ventricle, yet much of our understanding of disease progression relies on rodent studies because opportunities for investigation in humans are limited. We integrated plasma proteomic, metabolomic, and lipidomic profiles from 402 patients with PAH and 76 healthy controls using latent-network discovery and graphical modeling, with validation in an external cohort and comparison with human and bovine tissue. PAH was associated with broad molecular remodeling across peptides, metabolites, and lipids, with multiple multi-Omic programs converging on disruptions in the TCA cycle. Of note, elevated 2-hydroxyglutarate distinguished PAH across etiologies and comorbidity profiles, was associated with survival and treatment intensity, and was replicated externally. Related TCA-cycle changes were also observed in pulmonary vascular stromal and immune cells. These findings reveal coordinated remodeling across multiple molecular axes and highlight plasma multi-omics as a bridge between clinical phenotypes and tissue-level metabolic reprogramming.

## Highlights

• Latent factor analysis identified interacting multi-omics programs in PAH
• Dysregulated programs include TCA cycle, complement, coagulation, and ceramide metabolism
• 2-hydroxyglutarate distinguishes PAH from controls and predicts survival
• PAH plasma signatures mirror metabolic remodeling in pulmonary vascular cells

## Graphical abstract

**Figure:**
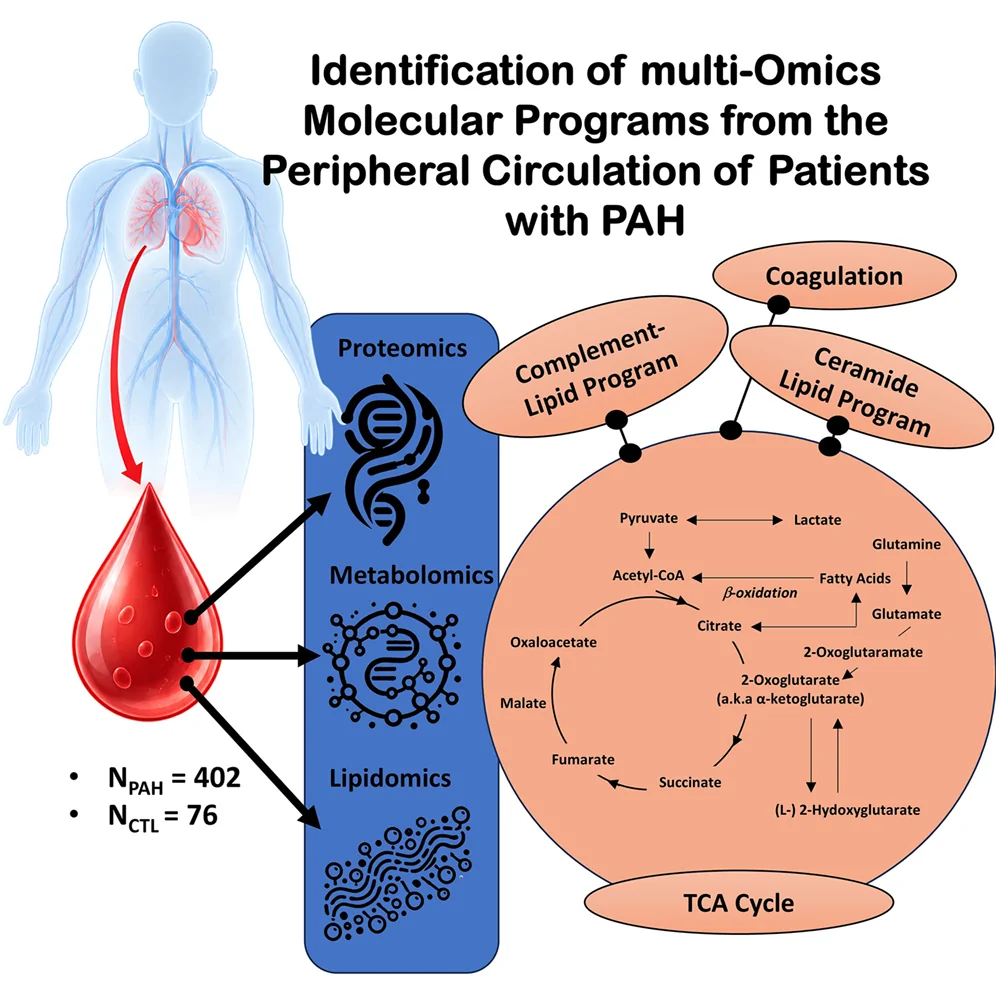

## Teaser

Health sciences

## Introduction

Pulmonary arterial hypertension (PAH) is a rare, progressive cardiopulmonary disease with no cure.^1^ Limited access to human lung tissue has forced reliance on preclinical animal models which, despite providing important biological insight, fail to capture disease heterogeneity and underlying causes critical for therapy development and outcome prediction.^2^^,^^3^^,^^4^^,^^5^^,^^6^ Although some features translate to humans,^7^ extrapolation from rodents remains limited,^8^ underscoring the importance of studying circulating factors in patient blood.

Biomarker discovery is challenged by both the large number of potential targets and uncertainty about relevant pathways. Blood-based studies have revealed transcriptomic and inflammatory heterogeneity not observable in animal models,^4^^,^^5^^,^^9^ highlighting the value of circulating markers. However, most investigations focus on a single omics layer—proteins, metabolites, or lipids—restricting their ability to capture integrated molecular networks and cross-domain interactions that may drive disease progression.^10^^,^^11^^,^^12^^,^^13^^,^^14^^,^^15^^,^^16^^,^^17^

Multi-omics strategies that simultaneously profile proteins, metabolites, and lipids offer a more comprehensive view of PAH biology. To date, no study has applied an untargeted analysis across all three domains within a single PAH cohort. Integrating these data with advanced machine-learning methods enables identification of key molecules and their interactions, revealing hidden drivers of disease.^3^

We hypothesized that unbiased integration of the proteome, metabolome, and lipidome would uncover cross-domain interactions not detectable with single-omics approaches, exposing coordinated biochemical networks and novel disease-relevant signatures in PAH.

## Results

Table 1 shows a breakdown of the demographic data for patients with CTL and PAH included in the study (additional data available in Data S1). Within patients with PAH, Kaplan-Meier curves (not shown) revealed statistical differences (*p* < 0.05) in survival based on etiology, but no differences based on sex or ethnicity. There were also no differences in survival based on the number of concurrent PAH-specific treatments (e.g., treatment naive vs. three PAH-specific treatments), and no relationship between the time since diagnosis and the number of PAH related therapies.

**Table 1:** Study cohort demographics in patients with CTL and PAH

	PAH	CTL	*p* value
N	402	76	–
Age (years ±SD)	52.3 ± 15.7	55.5 ± 16.2	0.11
% female patients	75%	61%	0.02
Race			
White	211	57	0.02
Black	30	4	
Asian or Pacific Islander	64	9	
Hispanic	61	4	
Native American	2	0	
Other	31	2	
Unknown	3	0	
Etiology			
Congenital heart disease, CHD	51	–	–
Connective tissue disease, CTD	122	–	–
Chronic thromboembolic PH, CTPH	5	–	–
Drug and toxin, D&T	84	–	–
Hereditary PAH, HPAH	3	–	–
Idiopathic PAH, IPAH	112	–	–
Portopulmonary hypertension, PoPH	25	–	–
Hemodynamics			
mPAP (mmHg)	48.3 ± 15.2	–	–
PVR (Wood units)	10.7 ± 5.92	–	–
Cardiac index ((L/min)/m^2^)	2.11 ± 0.61	–	–
Mean right atrial pressure (mmHg)	9.14 ± 5.16	–	–
PCWP (mmHg)	11.7 ± 4.57	–	–
Timing from diagnosis (years ±SD)	3.29 ± 5.94	–	–
Therapy			
Treatment naive	180 (45%)	–	–
Monotherapy	100 (25%)	–	–
Dual therapy	83 (21%)	–	–
Triple therapy	39 (10%)	–	–

Continuous data are compared using a *t* test and categorical data by ***χ***^2^ test.

### Differential abundance patterns across metabolomic, lipidomic, and proteomic domains between patients with PAH and CTL subjects

We identified 832 peptides from 291 proteins (59.9% peptides upregulated), 45 metabolites (77.8% upregulated), and 222 lipids (69.8% upregulated) that were significantly deregulated in PAH versus CTL subjects. These domain-specific patterns are summarized by volcano plots in Figures 1A–1C.

**Figure 1:**
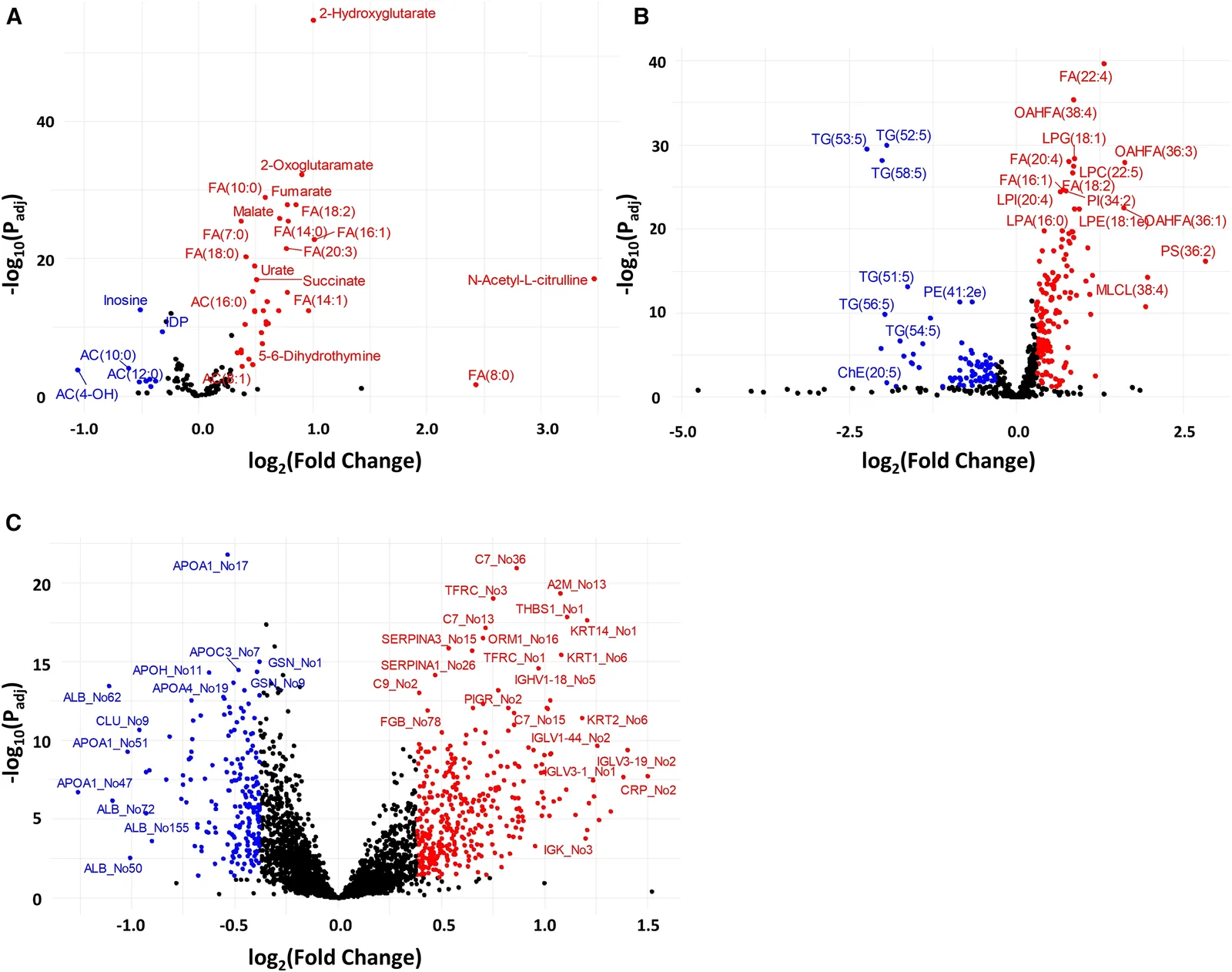
Differential expression results of plasma proteomic, metabolomic, and lipidomic Volcano plots show differentially abundant metabolites (A), lipids (B), and peptides (C) in 402 PAH versus 76 CTL subjects. Each numbered peptide (e.g., APOA1_No17) can be cross-referenced in Table S1. “Pval” denotes the adjusted *p* value following Benjamini-Hochberg false discovery rate correction. Features with |log2(fold change)| > 0.3 and adjusted *p* < 0.05 are shown in blue (decreased) or red (increased) in PAH relative to CTL.

We analyzed peptides rather than proteins because peptide abundance provides a more unprocessed measure of the data and captures signatures related to post-translational modifications. Table S1 lists all peptide names with corresponding sequences and descriptions. However, we also examined protein abundance to confirm consistency with prior studies, which identified established PAH markers (Figure S1), including activin (INHBE; *p* = 2.24e−7; 25% increase), immunoglobulins, complement proteins (C7, C9, CRP), platelet activation (PPBP), and protease inhibitors (SERPINs). Metabolomic and lipidomic findings aligned with prior studies, including markers of hypoxia activation (e.g., 2-hydroxyglutarate^18^), urate,^5^^,^^19^ malate,^20^ and disrupted fatty acid metabolism.^14^

We performed a peptide-level directionality concordance analysis as a quality-control assessment. Among proteins represented by at least three measured peptides, we first applied a conservative definition in which a protein was considered directionally concordant only if more than half of its evaluable peptides showed statistically significant changes in the same direction. Peptides without available quantitative or statistical data were excluded from the denominator. Under this definition, 21% of proteins met the concordance criterion. Because this approach requires peptide-level statistical significance, it likely underestimates directional consistency, particularly for proteins with missing peptide measurements or peptides showing modest but directionally consistent changes. We therefore interpret this analysis as a conservative peptide-level concordance assessment rather than as a formal protein-inference criterion. As a less stringent analysis, we also evaluated concordance using only the sign of the peptide log2 fold-change, without requiring peptide-level statistical significance. Under this less conservative definition, 96.4% of proteins met the concordance criterion, indicating that most proteins showed majority peptide-level directional agreement when statistical significance was not imposed at the individual peptide level.

### Discovery of multi-omic molecular programs of PAH

To explore how PAH predictive molecular candidates interact with other compounds across the three omics domains, we employed latent variable discovery (SLIDE analysis^21^). Each latent variable represents a coordinated molecular “program” consisting of co-regulated circulating compounds.

Figure 2A displays the resulting network of latent variables and their contributions to PAH prediction. Salmon-colored nodes represent stand-alone latent variables (predictive of PAH on their own) and gray nodes represent interacting latent variables (help predict PAH when interacting with a stand-alone latent variable). Connections between nodes indicate these interactions.

**Figure 2:**
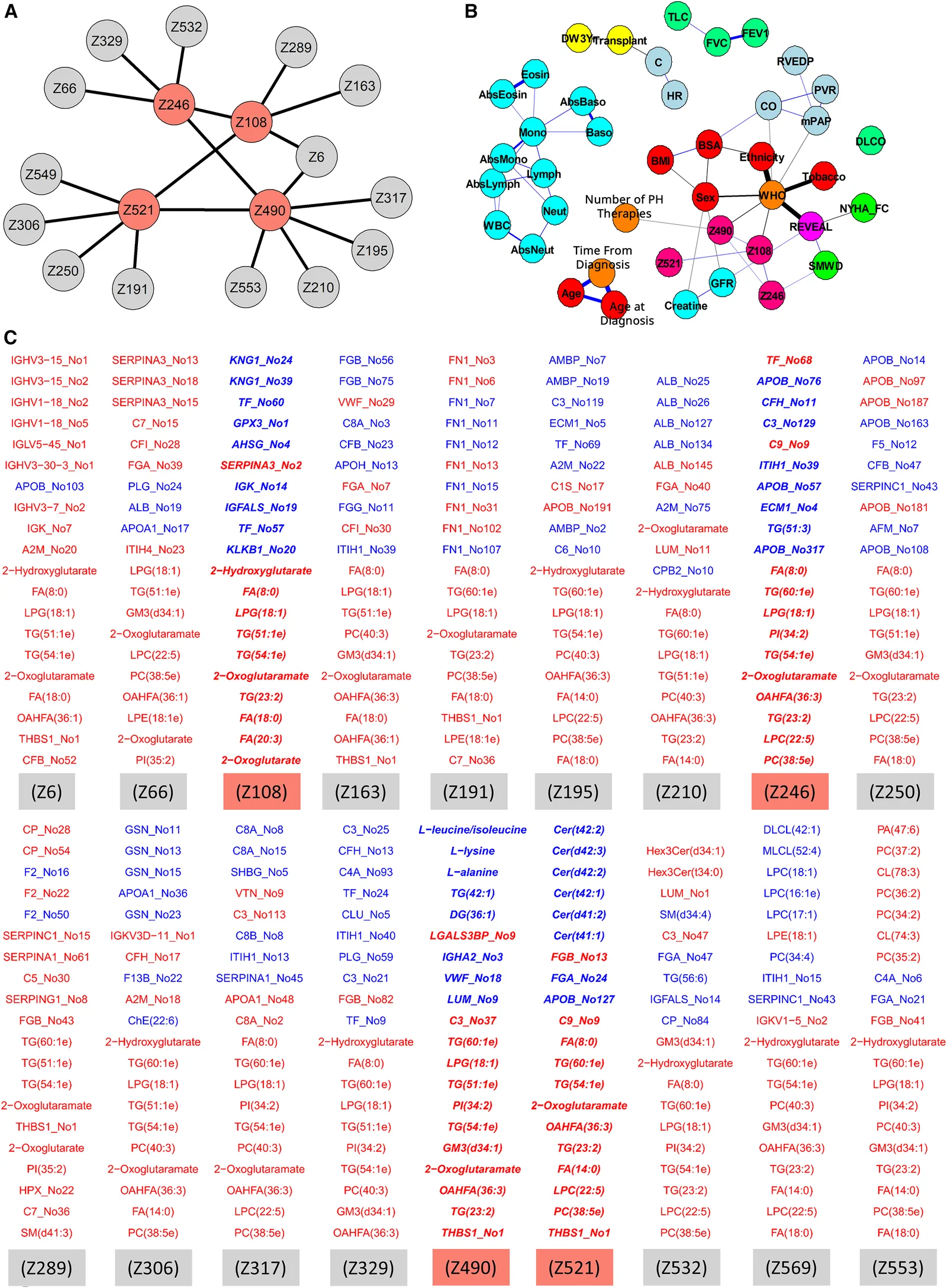
Latent network analysis results (A) Network shows interactions between underlying drivers of PAH. Salmon-colored nodes represent latent factors that independently classify PAH, whereas gray nodes indicate interacting latent factors. (B) Association network linking stand-alone latent factors identified in (A) with clinical markers, estimated using MGM. The network is displayed using a Fruchterman-Reingold layout. Edges are undirected and do not imply causality or direction of effect; edge widths represent rank-transformed association strength. Edge colors indicate association type: blue and red edges represent positive and negative associations between continuous variables, respectively, and black edges represent associations involving categorical variables. Nodes are colored by domain: red = demographics; orange = disease history and treatment; yellow = outcomes; green = exercise and functional measures; mint green = lung function; cyan = clinical blood and urine markers; light blue = RHC/hemodynamics; magenta = disease severity/risk; hot pink = stand-alone latent factors. (C) Stand-alone latent programs are bolded, with red indicating increased abundance and blue indicating decreased abundance in PAH.

To ensure that the identified latent networks reflect true disease-associated molecular structure rather than overfitting to noise, SLIDE’s standard validation procedures include repeated cross-validation and comparison of model performance against randomized and knockoff controls. True latent networks consistently demonstrated superior discrimination of PAH relative to both fully randomized factors and knockoff variables, supporting that SLIDE identifies real, disease-related molecular patterns. Full methodological details are provided in Methods S1, and validation analyses are shown in Figure S2.

To determine how the latent space reflects patient pathobiology, we examined the relationships between clinical domains and SLIDE-derived molecular programs (Figure 2B) using mixed graphical modeling. Hemodynamics, lung function, laboratory values, and demographics behave as largely independent information blocks with little shared variance, with lung function and laboratory measures showing minimal association with catheterization data or each other. Only a small set of variables, primarily select catheterization metrics and kidney-related labs (e.g., GFR), formed an interconnected central hub with nodes reflecting disease severity, exercise tolerance, and etiology. Interestingly, the SLIDE latent variables converged on this same hub, rather than an isolated molecular signal such as clinical blood and urine markers, aligning multi-omic programs with the few clinical features carrying meaningful prognostic or pathophysiologic information (see Figure S3 and Methods S1 for additional information on the prognostic signal within the latent space).

Figure 2C displays the molecular composition of each SLIDE-derived program. Each latent variable is represented by 20 compounds: the top 10 are ranked by loading and therefore represent the compounds that most strongly define the program, whereas the bottom 10 are ranked by univariate AUC for distinguishing PAH from CTL. Red indicates higher abundance in PAH, while blue indicates lower abundance.

Figure 3 shows heatmaps of the compounds represented in each stand-alone program. For each program, we show the 10 compounds with the highest absolute feature loadings within the program and the 10 compounds with the strongest univariate discrimination of PAH. For each selected compound, the heatmaps display measured abundance across patients, with patients organized by PAH etiology. Across programs, these abundance patterns did not cluster clearly by etiology, suggesting that the stand-alone programs do not reflect etiology-specific molecular signatures. Notably, although the program-level scores were predictive of PAH status, many of the individual high-loading compounds within these programs were not strongly predictive on their own. This suggests that the discriminatory signal captured by the stand-alone programs arises from coordinated multivariate structure rather than from single dominant biomarkers.

**Figure 3:**
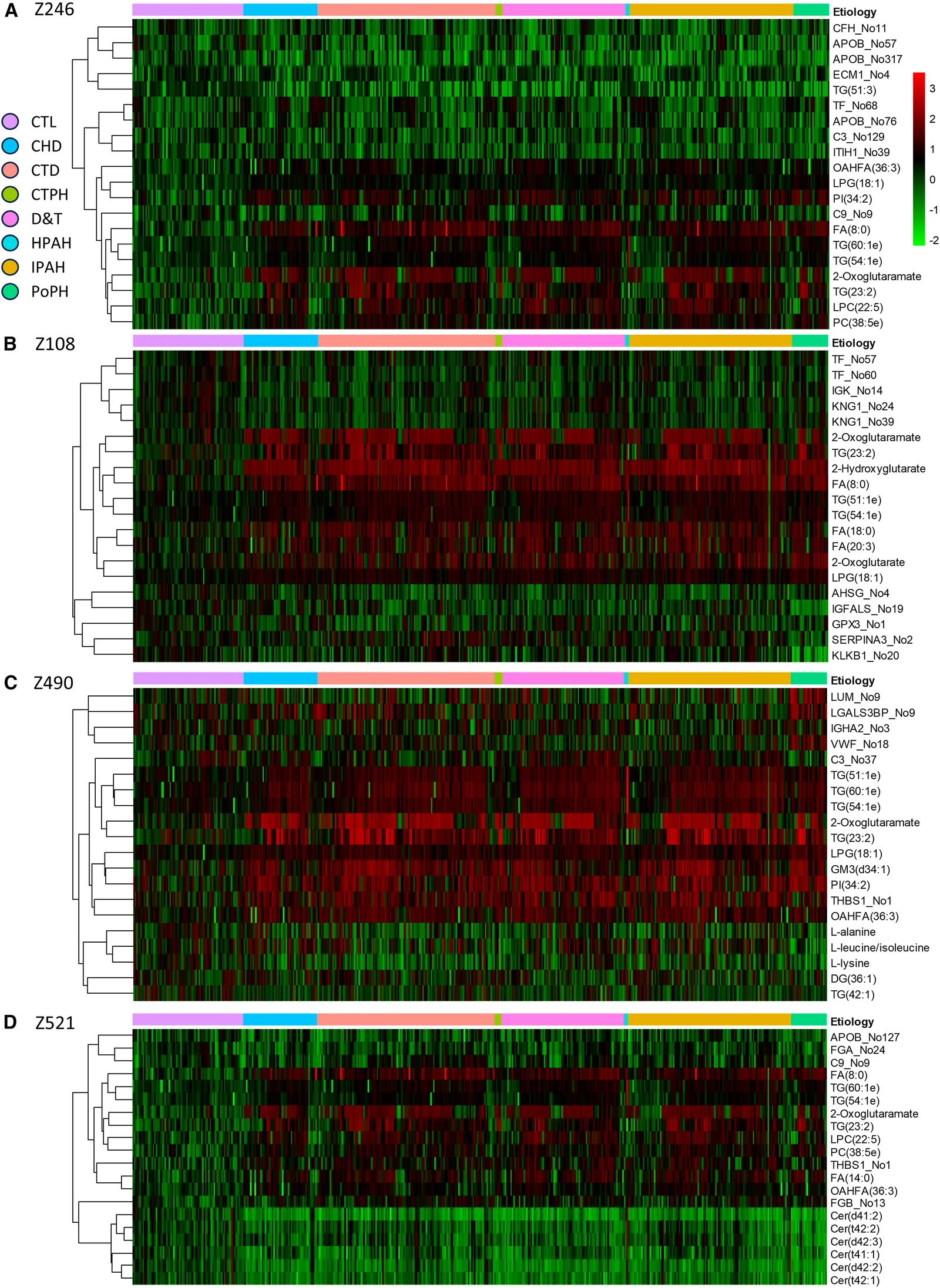
Molecular patterns of the latent networks (A–D) Heatmaps show the molecular features within the four stand-alone latent networks. The annotation bar groups subjects by CTLs (left) and by PAH etiology.

Overall, our analysis identified four stand-alone latent variables. Z108 consisted of high-weight peptide signals derived from kininogen (KNG1), serotransferrin (TF), and related plasma proteins involved in coagulation and protease regulation. In CTL subjects, these peptides are largely uncoupled from metabolism. In PAH, however, they become tightly linked to high-discrimination TCA-cycle intermediates, including 2-oxoglutarate, 2-oxoglutaramate, and 2-hydroxyglutarate (see Figure 4).

**Figure 4:**
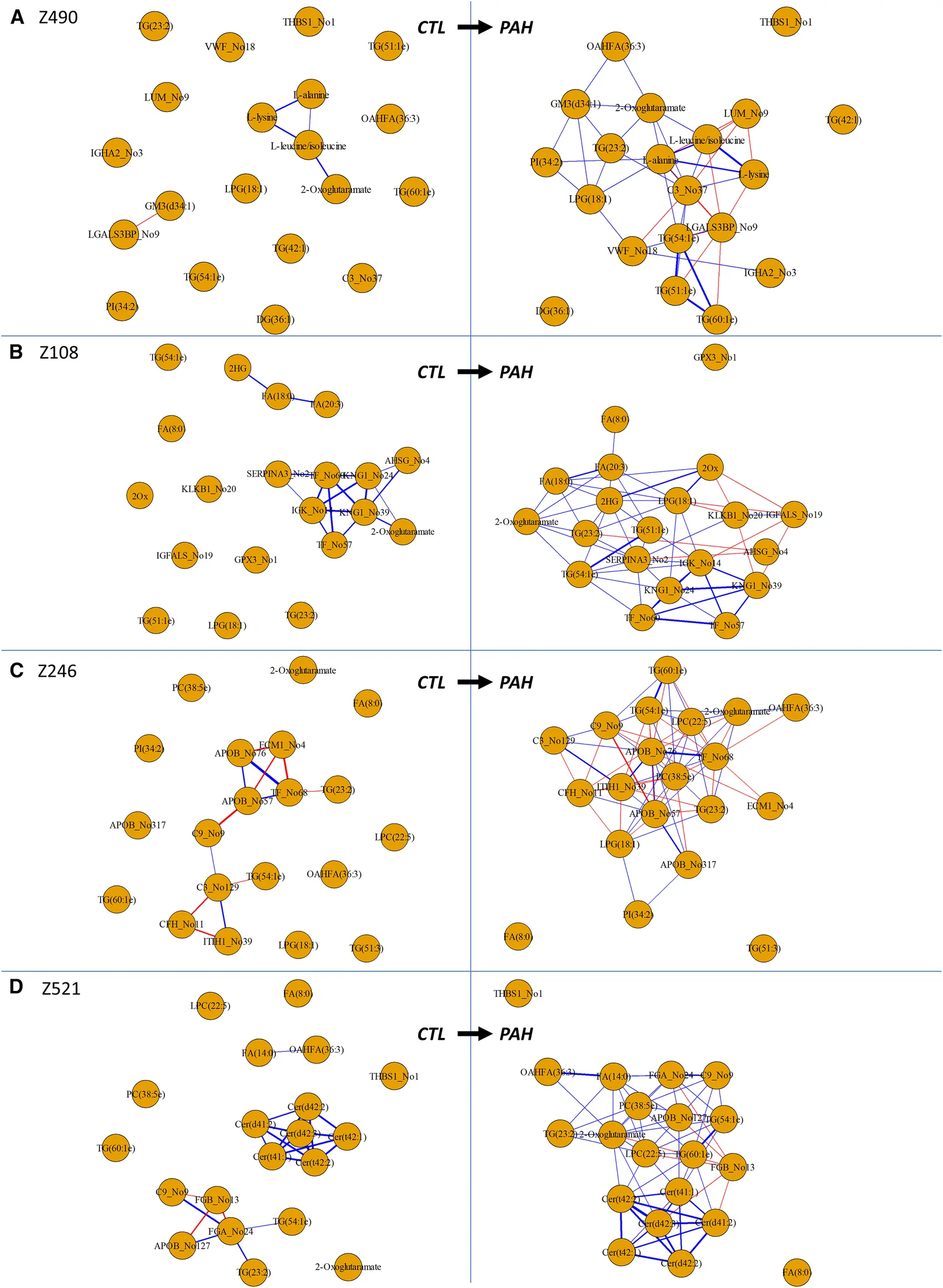
Correlation structure of each latent program (A–D) Pairwise correlation networks of features within stand-alone programs in CTLs (left) and PAH (right) subjects. Edges indicate significant correlations (*α* = 0.001), with blue denoting positive and red denoting negative correlations; edge width is proportional to correlation magnitude.

Z246 was another stand-alone latent variable and represented a mixed peptide-lipid program, with prominent contributions from complement-associated peptides, including C3, C9, and CFH, as well as lipid-transport and extracellular matrix-associated proteins (see Figure 2C). Within the overall SLIDE network, Z246 indirectly connects to Z6, a distinct latent factor enriched for immunoglobulin heavy-chain variable region peptides (including IGHV1 and IGHV3). Although complement-associated peptides and immunoglobulin-derived signals were not directly linked, their indirect connection within the global network structure is consistent with patterns we previously reported.^16^

Z490 and Z521 captured separate metabolic and lipid-associated programs. Z490 was defined by contributions from amino acids and lipid-associated features, whereas Z521 was dominated by ceramide species and related lipids. Ceramide species contributing to Z521 were lower in the peripheral circulation of patients with PAH compared with CTL subjects, making Z521 one of the most coherent lipid-dominated latent factors identified in the analysis.

### The TCA cycle in PAH

A repeated theme of the latent factor analysis pointed to a glutamine-driven replenishment of the TCA cycle under metabolic stress (see Figure 5A). The coordinated elevation of 2-oxoglutaramate (Figure 5B), 2-oxoglutarate (Figure 5B), and 2-hydroxyglutarate (Figure 6) supports increased glutamine-dependent anaplerosis, consistent with a state in which cells rely on glutamine to sustain TCA activity when mitochondrial function is impaired. Other TCA intermediates, including Succinate, Fumarate, and Malate, are also consistently elevated in PAH (see Figure 5B).

**Figure 5:**
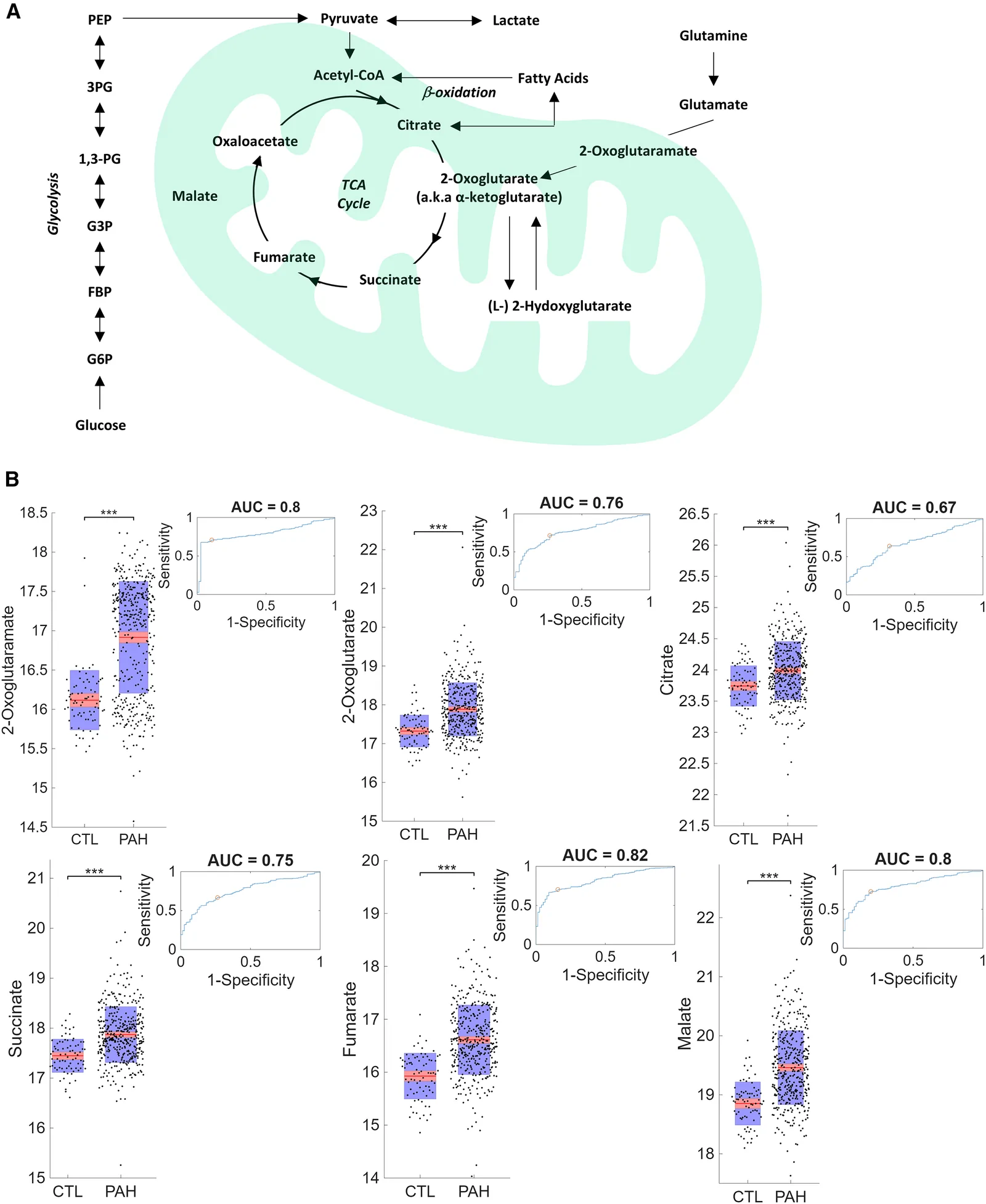
Circulating expression of TCA cycle biomarkers (A) Schematic illustrating glutamine-driven anaplerosis of the tricarboxylic acid (TCA) cycle and hypoxia-associated diversion of 2-oxoglutarate toward (L-)2-hydroxyglutarate. (B) Plots show circulating levels of TCA-cycle intermediates in CTL (*N* = 76) and PAH (*N* = 402) subjects, with corresponding receiver operating characteristic curves inset for each metabolite. Area under the curve (AUC) values indicate classification performance for PAH. Statistical significance (*p* < 0.001) between groups is indicated by asterisks (∗∗∗). Additional statistical details are available in Data S1 (STAR Methods). Comparative plots show individual subjects (black dots), the mean (red line), the 95% confidence interval of the mean (red shading), and ±1 standard deviation (blue shading).

**Figure 6:**
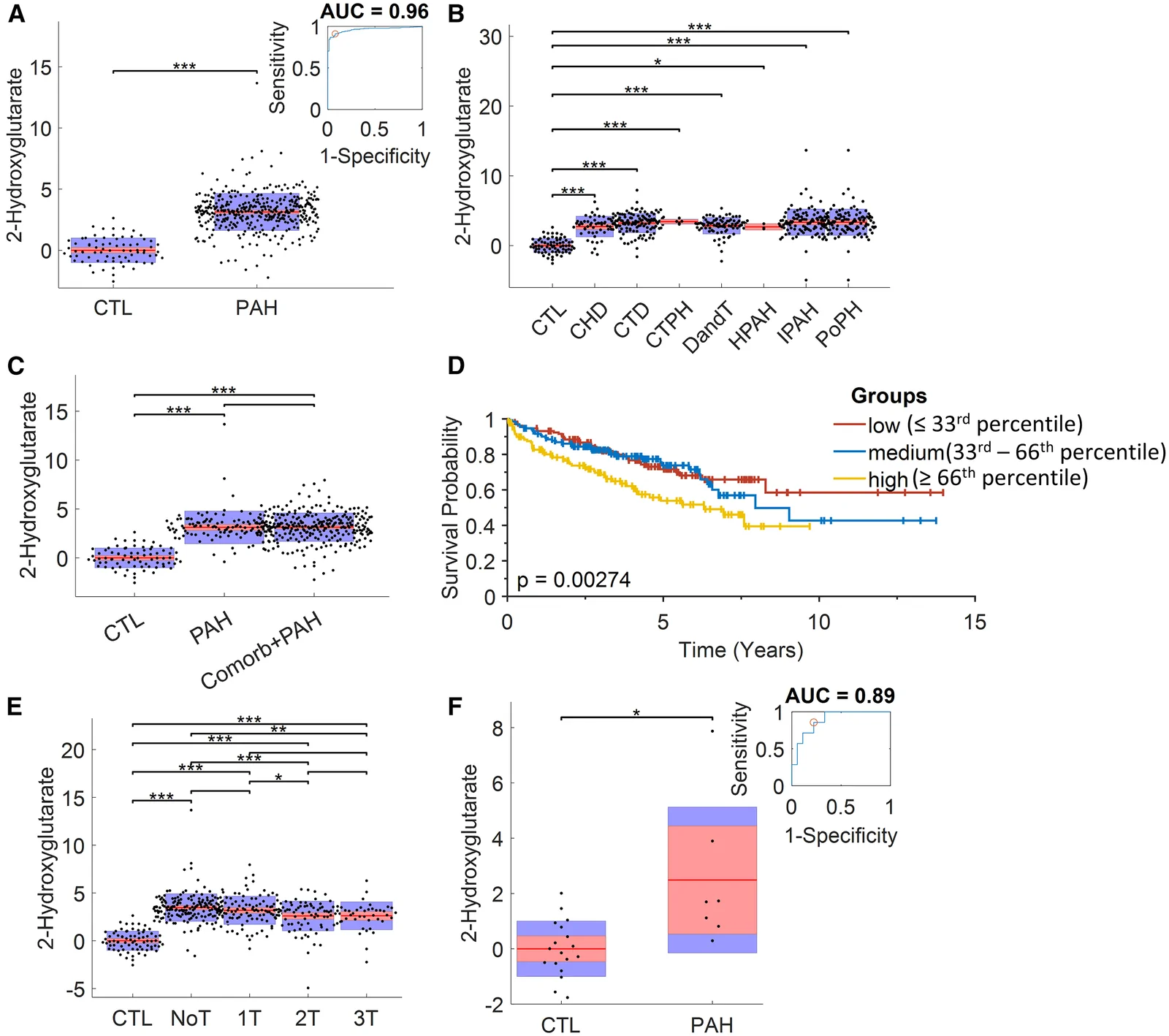
Circulating 2-hydroxyglutarate in PAH (A) 2-hydroxyglutarate compared between CTL and PAH subjects. (B) 2-hydroxyglutarate levels compared between CTLs and sub-etiologies of PAH. (C) 2-hydroxyglutarate levels compared between patients with CTLs and PAH with and without comorbidities (defined in text). (D) Survival curve for all 402 patients with PAH after being stratified based on low (bottom 33^rd^ percentile), medium, or high (upper 67^th^ percentile) plasma 2-hydroxyglutarate levels. (E) 2-hydroxyglutarate levels compared between patients with CTLs and PAH categorized by the number of concurrent PAH-specific treatments. NoT = treatment-naïve patients; 1T = receiving single PAH treatment; 2T = dual PAH treatment; and 3T = triple-combination PAH treatment. (F) 2-hydroxyglutarate levels compared between patients with CTL and PH in the external validation cohort. *p* values were computed by *t* test (Welch’s *t* test in cases of unequal variance) (∗*p* < 0.05; ∗∗*p* < 1e−2; ∗∗∗*p* < 1e−3). Additional statistical details are available in Data S1 (STAR Methods). Note: Comparative plots show individual subjects (black dots), the mean (red line), the 95% confidence interval of the mean (red shading), and ±1 standard deviation (blue shading).

Interestingly, the circulating marker of the TCA cycle that stood out in our data was 2-hydroxyglutarate. It differentiated PAH from CTL subjects with 91% sensitivity and 92% specificity (Figure 6A). It was consistently elevated across all PAH subtypes (Figure 6B) and in patients with PAH both with and without comorbidities (Figure 6C). Comorbidities were defined as pulmonary, sleep-related, thyroid, congenital, or atherosclerotic cardiovascular conditions, including COPD, interstitial lung disease, asthma, sleep-disordered breathing, thyroid disease, congenital heart disease, coronary or peripheral arterial disease, and active tobacco use.

To examine the prognostic relevance of 2-hydroxyglutarate, patients were stratified into low (lower 33rd percentile), medium, and high (upper 67th percentile) groups. Those in the highest group showed a statistically significant reduction in survival (Figure 6D). We also evaluated whether PAH-specific treatment itself contributed to elevated 2-hydroxyglutarate. Instead, we found the opposite: levels were highest in treatment-naïve patients and decreased progressively with increasing treatment intensity (Figure 6E).

Elevated 2-hydroxyglutarate levels were also observed in an independent external validation cohort (Figure 6F) from the Mass General Brigham health care system. Mixed venous plasma was obtained under Mass General Brigham IRB approval (2011p000272) from patients with PAH at diagnostic right heart catheterization and from CTL subjects with normal pulmonary hemodynamics during invasive cardiopulmonary exercise testing. Despite the small sample size (18 CTL, 7 PAH), the same trend toward elevated 2-hydroxyglutarate in PAH was observed.

Figure 7A illustrates TCA cycle dynamics, now including the conversion of 2-oxoglutarate to 2-hydroxyglutarate. Figures 7B and 7C show re-analysis of the metabolic state of human (b) and bovine (c) CTL and PH fibroblasts,^22^ obtained from distal pulmonary arteries (diameter < 2 mm), which reveals a pattern closely mirroring our circulating metabolomic signature, including coordinated upregulation of TCA intermediates. The bovine fibroblasts were derived exclusively from male Holstein calves exposed to hypobaric hypoxia for 2 weeks beginning at one day of age. The human fibroblast cohort included both male and female donors. The three control subjects used for metabolomic analysis included two males and one female (age = 29.3 ± 11.5 years old), while the three IPAH subjects included two females and one male (age = 24.4 ± 11.5 years old).

**Figure 7:**
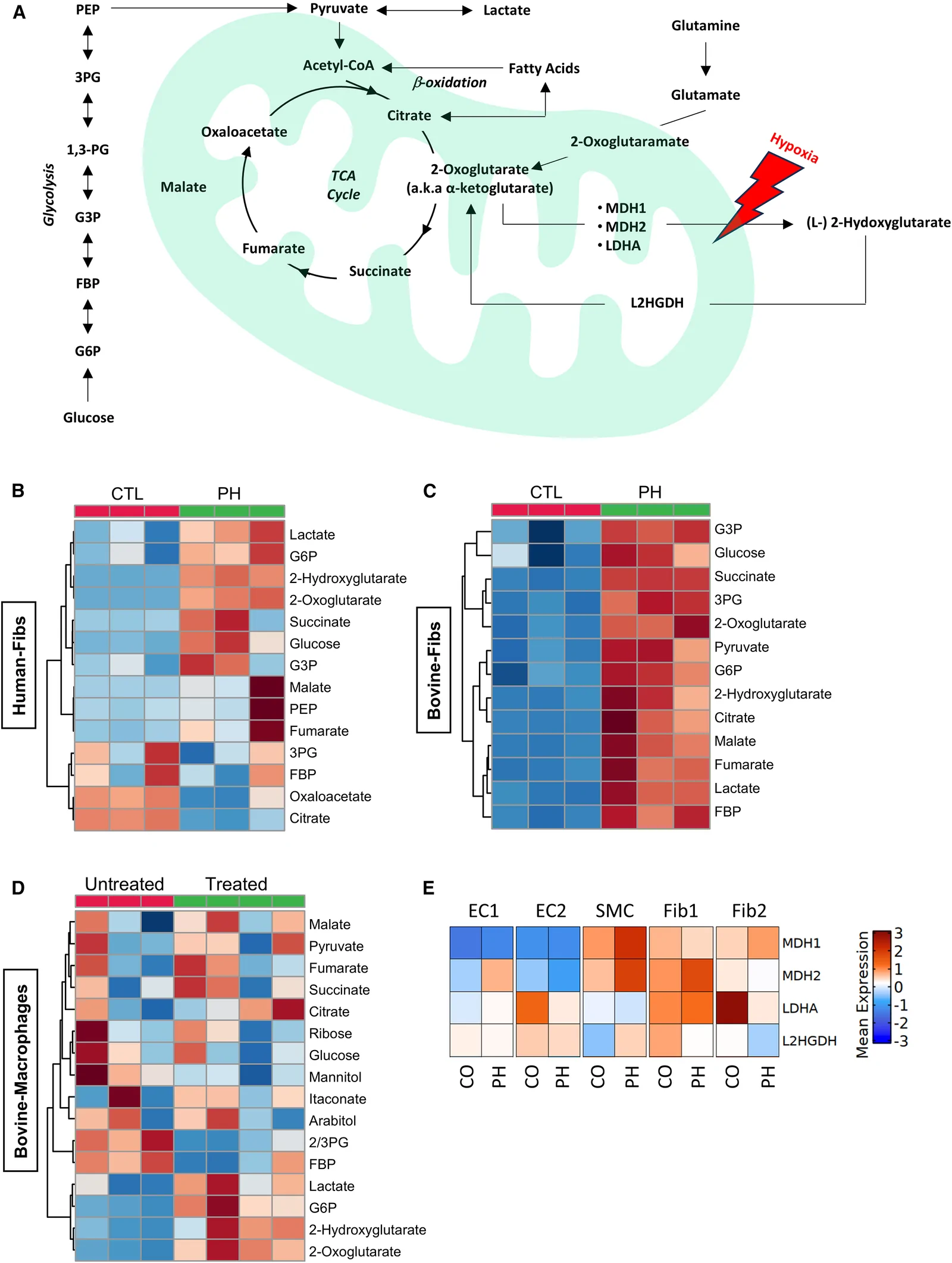
Circulating TCA remodeling is mirrored by metabolic changes in pulmonary vascular and immune cells (A) Schematic illustrating glutamine-driven anaplerosis of the tricarboxylic acid (TCA) cycle and hypoxia-associated diversion of 2-oxoglutarate toward L-2-hydroxyglutarate via the promiscuous activity of MDH1, MDH2, and LDHA, with L2HGDH catalyzing the reverse reaction. (B and C) Heatmaps obtained by re-analysis of data obtained from ref.^22^ of TCA-cycle and glycolytic intermediates in human and bovine pulmonary fibroblasts, respectively, comparing CTL and PH conditions. (D) Metabolic profiling of bone marrow-derived macrophages treated with conditioned medium from PH fibroblasts, compared with untreated macrophages, demonstrating marked upregulation of 2-oxoglutarate and L-2-hydroxyglutarate. (E) Single-cell RNA sequencing analysis of stromal cells from distal pulmonary arteries of patients with pulmonary arterial hypertension (PAH; GSE210248) shows increased expression of MDH1 and MDH2 and reduced expression of L2HGDH in fibroblast and smooth muscle cell populations, favoring intracellular accumulation of L-2-hydroxyglutarate.

We and others have also established metabolic reprogramming as a key feature of macrophages in the PH vessel wall.^23^ Figure 7D shows altered metabolism in bone marrow-derived macrophages from a bovine (all males) PH model following treatment with conditioned medium from PH fibroblasts. Notably, both 2-oxoglutarate and 2-hydroxyglutarate are strongly upregulated.

Finally, L-2-hydroxyglutarate is derived from 2-oxoglutarate, promiscuously formed by activities of malate dehydrogenases 1 and 2 (MDH1 and MDH2), and lactate dehydrogenase A (LDHA) in hypoxic and acidic conditions. Cellular levels of L-2-hydroxyglutarate are normally constrained by L-2-hydroxyglutarate dehydrogenase (L2HGDH), which catalyzes its conversion back to 2-oxoglutarate (see Figure 7A). In single-cell RNA sequencing analysis of stromal cells within distal pulmonary arteries from humans with PAH^24^ (GSE210248), MDH1 and MDH2 were markedly upregulated specifically in smooth muscle cells and fibroblast populations (Figure 7E), while LDHA expression was not consistently increased. In parallel, L2HGDH expression was reduced, collectively favoring intracellular accumulation of L-2-hydroxyglutarate. Control samples were obtained from three male donors (age = 51.4 ± 11.6 years), whereas PAH samples were obtained from two male and one female donor (age = 49.5 ± 13.1 years).

## Discussion

We performed an integrated multi-omic analysis of plasma from 402 patients with PAH and 76 CTL subjects using unbiased metabolomic, lipidomic, and proteomic profiling, coupled with machine-learning approaches to identify coordinated disease-associated programs.

Many of the differentially abundant circulating molecules identified in our dataset recapitulate established PAH biology. These include complement and protease-regulatory proteins (e.g., A2M, APOA1, C7, C9, SERPINs, and immunoglobulin fragments^16^), metabolites linked to mitochondrial stress and hypoxia (e.g., 2-hydroxyglutarate, malate, fumarate, and 2-oxoglutarate^25^), and broad alterations in lipid species such as fatty acids and triglycerides.^14^ This concordance supports the biological validity of the circulating signal.

SLIDE analysis further showed that these molecules organize into interacting multi-omics programs rather than acting in isolation. A central feature of these programs was the emergence of coordinated relationships between TCA-cycle intermediates and other disease-associated molecules, which were largely absent in CTL but prominent in PAH.

TCA-cycle intermediates were uniformly elevated in PAH, indicating a coordinated shift in central carbon metabolism. Among these, 2-hydroxyglutarate showed robust elevation across disease subtypes and strong associations with clinical outcomes, implicating altered TCA-cycle flux and hypoxia-linked metabolic remodeling as core features of the PAH circulating phenotype.

### Emergent multi-omic organization in PAH revealed by latent factor analysis

To identify coordinated multi-omics programs spanning the proteome, metabolome, and lipidome in PAH, we applied latent factor discovery.^21^^,^^26^ Although many traditional machine-learning approaches focus on identifying individual markers with strong predictive power, SLIDE is designed to uncover coordinated molecular programs in PAH, which consist of sets of features whose collective behavior is informative even when the individual components are weak or non-predictive on their own.

A key feature of one stand-alone program was the central contribution of KNG1-derived peptides. Rather than behaving as an isolated plasma protein signal, KNG1 clustered with TF and related coagulation/protease-regulatory peptides and became linked to TCA-cycle intermediates specifically in PAH. This coupling was not apparent in CTL subjects, suggesting that the KNG1-associated signal reflects a disease-specific interaction between plasma protease/coagulation biology and altered mitochondrial metabolism. This pattern indicates disease-specific engagement of hypoxia- and mitochondria-associated metabolism. Notably, although some prior studies have shown that KNG1 expression is increased within the lungs of multiple PAH rodent models,^27^^,^^28^ circulating KNG1 has been reported to be decreased.^28^ Our data are consistent with this finding: KNG1-derived peptides within Z108 are reduced in plasma and participate in a coordinated program with metabolic intermediates that emerges specifically in PAH, highlighting a divergence between tissue-level accumulation and systemic depletion previously characterized in rodent models.

Other complement-, immunoglobulin-, amino acid-, and lipid-associated latent factors indicate that PAH is characterized by coordinated remodeling across several partially distinct molecular axes rather than a single dominant circulating signature. Importantly, the lipid-associated programs were not limited to broad lipid abundance changes but included a coherent ceramide (Cer)-related signal that was reduced in plasma. This coordinated decrease in circulating Cer species is notable because prior work has shown that Cer production can be locally increased or dysregulated in pulmonary endothelial cells, where it contributes to vascular injury through intracellular and paracrine mechanisms.^29^ Thus, reduced Cer levels in plasma do not necessarily indicate reduced Cer signaling within the pulmonary vascular compartment. Instead, these findings suggest a potential divergence between circulating lipid abundance and tissue-level lipid activity in PAH, which requires further investigation.

### TCA-cycle reprogramming as a central feature of PAH molecular networks

A striking feature of our analysis is the remarkably uniform upregulation of TCA cycle intermediates in PAH, observed consistently across metabolites rather than confined to a single node of the pathway, with each intermediate showing strong individual discrimination between PAH and CTL subjects. Compounds including 2-oxoglutarate, 2-oxoglutaramate, 2-hydroxyglutarate, succinate, fumarate, citrate, and malate are all elevated in PAH subjects, consistent with a coordinated shift in central carbon metabolism. Beyond abundance, these intermediates show pronounced network convergence in PAH: metabolites that are relatively independent in CTL subjects become tightly co-regulated and embedded within shared multi-omic programs in disease. This pattern is consistent with prior reports in PAH lung tissue describing hypoxia-associated TCA-cycle dysfunction and metabolic rewiring toward glutamine anaplerosis.^19^^,^^30^^,^^31^^,^^32^^,^^33^ Although citrate is often interpreted as reflecting pyruvate-derived carbon entry, its elevation here may instead reflect glutamine-driven anaplerosis coupled to TCA bottlenecking under mitochondrial stress, rather than increased glycolytic flux. Glutamine represents a key substrate for the synthesis of glutamate via glutaminolysis, a pathway where cells convert glutamine → glutamate → 2-oxoglutarate (a.k.a α-ketoglutarate).

One particularly striking metabolite is 2-hydroxyglutarate. Mechanistically, 2-hydroxyglutarate promotes stabilization and enhanced activity of hypoxia-inducible factors (HIFs) by interfering with prolyl hydroxylase activity, thereby reinforcing hypoxia signaling; HIFs are transcription factors activated under low-oxygen conditions and are well known to contribute to the development and progression of PAH.^34^^,^^35^^,^^36^ The strength and consistency of the 2-hydroxyglutarate signal in our dataset, replicated in a small external validation cohort, suggests that it may capture a core metabolic axis of PAH, rather than a subtype-specific feature, with potential diagnostic and prognostic application.

2-hydroxyglutarate has been described as an oncometabolite and has been found to elicit significant epigenetic changes that can contribute to cancer or cancer-like phenotype.^37^ Additionally it should be noted that other TCA cycle intermediates such as succinate, 2-oxoglutarate, and fumarate can also regulate histone and DNA methylation.^38^ Furthermore, in addition to the effects of 2-oxoglutarate on prolyl hydroxylase, it has also been reported that supraphysiologic levels of succinate and fumarate can replace 2-oxoglutarate as a mock substrate for prolyl hydroxylase, thereby preventing HIF1 hydroxylation and promoting its stabilization and thereby further enhancing the Warburg effect.

Importantly, these circulating signatures are mirrored by tissue-level metabolic changes within the pulmonary vasculature. Reanalysis of published metabolomic and transcriptomic datasets from pulmonary arterial fibroblasts and smooth muscle cells isolated from bovine PH and human PAH lungs reveals coordinated upregulation of TCA-cycle intermediates and related metabolic pathways, closely paralleling the observed changes in the circulation of patients with PAH. Similar metabolic reprogramming has also been reported in macrophages residing within the PAH vessel wall, where elevated levels of 2-oxoglutarate and 2-hydroxyglutarate accompany hypoxia and inflammation associated activation states.^23^ Together, these findings support the interpretation that the uniform elevation and network convergence of TCA-cycle intermediates observed in plasma reflect cell-intrinsic metabolic remodeling within diseased pulmonary vascular tissues, rather than a nonspecific systemic epiphenomenon.

Taken together, these plasma-based findings strongly suggest that TCA-cycle reprogramming in PAH is not merely a systemic epiphenomenon but reflects cell-intrinsic metabolic remodeling within the pulmonary vascular niche. Consistent with this idea, both our own data and prior studies demonstrate a robust enrichment of TCA-cycle intermediates and associated metabolic pathways within stromal and immune cells isolated from the PAH vessel wall.^22^^,^^39^^,^^40^^,^^41^

### Limitations of the study

Some patients had documented COPD or CTEPH, which could classify them as WHO group 3 or 4 PH. However, because their hemodynamics were disproportionate to lung disease and PAH was the official clinical diagnosis, they were analyzed as PAH, with COPD or CTEPH treated as secondary conditions.

The MS-based proteomics approach used in this study did not employ enrichment techniques and therefore had limited sensitivity for low-abundance molecules. As an example, lactate was excluded from downstream analyses due to poor signal-to-noise ratio and technical variability. Although exploratory inspection suggested higher lactate in PAH, consistent with other TCA-cycle intermediates and prior literature,^18^ conflicting reports of decreased lactate^25^ may partly explain this instability. Consequently, other low-abundance signaling molecules, including cytokines and members of the TGF-beta pathway, were likely not captured.

Finally, the validation cohort was relatively small, limiting our ability to fully evaluate 2-hydroxyglutarate, and other potential candidates as biomarkers.

## Resource availability

### Lead contact

Further information and requests for resources should be directed to and will be fulfilled by the lead contact, Vitaly O. Kheyfets (vitaly.kheyfets@cuanschutz.edu).

### Materials availability

This study did not generate new unique reagents or materials.

### Data and code availability

• Data: The datasets analyzed in this study are publicly available at the repositories and accession numbers specified in the manuscript. The mass spectrometry proteomics data have been deposited in the ProteomeXchange Consortium via the PRIDE partner repository with the dataset identifier PXD083317 (https://www.ebi.ac.uk/pride/archive/projects/PXD083317). The plasma metabolomics data have been deposited in Metabolomics Workbench under PR003370 (https://dev.metabolomicsworkbench.org:22222/data/DRCCMetadata.php?Mode=Study&StudyID=ST005219&StudyType=MS&ResultType=1&Access=AwnW8130). Previously published datasets analyzed in this study are available as described in the original publications (https://doi.org/10.1161/CIRCULATIONAHA.117.028069; https://doi.org/10.3389/fimmu.2021.640718).
• Code: This paper does not report original code.
• Other: Any additional information required to reanalyze the data reported in this paper is available from the lead contact upon request.

## Acknowledgments

The authors used a large language model as a writing assistance tool during manuscript preparation. The tool was used solely to proofread text and to improve clarity and brevity. All scientific content, data analysis, interpretation, and conclusions were generated and verified by the authors, who take full responsibility for the integrity and accuracy of the work.

Funding: 10.13039/100000002NIH P01HL152961 (VOK, KRS, and KCH); 10.13039/100000002NIH R01HL152250 (VOK); K08HL128802 (WMO).

## Author contributions

V.O.K. performed the data analysis and wrote the manuscript. A.J.S. assisted with data analysis and manuscript preparation and coordinated sample and data compilation. H.Z., M.L., and C.-J.H. contributed to data analysis and interpretation. T.N. performed mass spectrometry analyses and contributed to data interpretation. M.A. and P.H. contributed to data analysis and interpretation. M.D., D.S., and I.S.L. performed mass spectrometry analyses. A.D., F.I.C., and K.C.H. performed mass spectrometry analyses and contributed to data interpretation. W.M.O. provided the validation cohort and contributed to data interpretation. R.T.Z. coordinated sample collection and data compilation, contributed to data analysis and interpretation, and assisted with manuscript preparation. K.R.S. conceptualized the study, contributed to data interpretation, and assisted with manuscript preparation.

## Declaration of interests

The authors declare no competing interests.

## STAR★Methods

### Key resources table

**Table:** 

REAGENT or RESOURCE	SOURCE	IDENTIFIER
**Biological samples**		

Human plasma, pulmonary arterial hypertension primary cohort (*n* = 402)	Stanford Pulmonary Hypertension Biobank	IRB #14083
Human plasma, healthy control primary cohort (*n* = 76)	Stanford Cardiovascular Institute Biomarker and Phenotype Core Laboratory; Stanford Healthy Aging Study	IRB #40869; IRB #20942
Human mixed venous plasma, external validation cohort (7 PAH; 18 controls)	Mass General Brigham	IRB #2011p000272

**Deposited data**		

Primary plasma mass spectrometry proteomics dataset	ProteomeXchange Consortium via PRIDE	PXD083317; https://www.ebi.ac.uk/pride/archive/projects/PXD083317
Primary plasma metabolomics dataset	Metabolomics Workbench	PR003370https://dev.metabolomicsworkbench.org:22222/data/DRCCMetadata.php?Mode=Study&StudyID=ST005219&Access=AwnW8130
Human pulmonary artery single-cell RNA-sequencing data used for reanalysis	NCBI Gene Expression Omnibus (Crnkovic et al.^24^)	GSE210248
Human and bovine pulmonary artery fibroblast metabolomics data used for reanalysis	Zhang et al.^22^	https://doi.org/10.1161/CIRCULATIONAHA.117.028069
Bovine bone marrow-derived macrophage metabolomics data used for reanalysis	Li et al.^23^	https://doi.org/10.3389/fimmu.2021.640718

**Software and algorithms**		

MATLAB R2021b	MathWorks	https://www.mathworks.com/products/matlab.html
R v4.2.2	R Foundation for Statistical Computing	https://www.r-project.org/
NormalyzerDE v1.13.2	Bioconductor/Computational Proteomics	https://doi.org/10.18129/B9.bioc.NormalyzerDE;https://bioconductor.org/packages/NormalyzerDE/
mgm R package	Jonas Haslbeck/CRAN	https://CRAN.R-project.org/package=mgm
SLIDE (Significant Latent Factor Interaction Discovery and Exploration)	Rahimikollu et al.,^21^/ Das Lab	https://github.com/jishnu-lab/SLIDE; https://doi.org/10.5281/zenodo.10159961

### Experiment study and participant details

In accordance with the Declaration of Helsinki, this study analyzed plasma from 402 PAH patients and 76 control (CTL) subjects evaluated at Stanford University (2008–2014). PAH samples were obtained from the Stanford Pulmonary Hypertension Biobank (IRB #14083) after informed consent and collected during fasting right heart catheterization. PAH was hemodynamically defined as mean pulmonary arterial pressure ≥25 mmHg, pulmonary vascular resistance ≥240 dyn s cm^−5^, and pulmonary arterial wedge pressure ≤15 mmHg; additional criteria are described in Sweatt et al.^4^ Participant age, sex, race, and other demographic information are reported in Table 1.

CTL samples (2009–2013) were obtained from the Stanford Cardiovascular Institute Biomarker and Phenotype Core Laboratory and the Stanford Healthy Aging study (IRB #40869; #20942) after written consent and clinical screening. Individuals with major cardiopulmonary or inflammatory disease, diabetes, recent infection, Alzheimer’s disease, smoking history, or body mass index >35 were excluded. Fasting peripheral venous blood was collected and processed identically to PAH samples.

### Method details

Plasma was flash frozen at collection at Stanford, stored at −40°C, and shipped without thawing to the University of Colorado (Aurora, CO), where all MS analyses were performed. Details on sample preparation, MS configuration, and identification libraries are provided in Methods S1.

### Quantification and statistical analysis

Data analysis was performed using MATLAB 2021b (Mathworks, MA) and R 4.2.2.

Untargeted proteomics, metabolomics, and lipidomics identified 15,781 candidate circulating biomarkers. Prior to integrated normalization, each omics layer was evaluated separately using PCA, density plots, and boxplots to assess sample-level distributions and identify potential outliers or layer-specific technical artifacts. Across peptides, metabolites, and lipids, these analyses did not show obvious isolated sample clusters or gross outlier-driven separation. Global adjustment was applied to reduce systematic biases (e.g., sample loading differences)^42^ and the normalizer library (NormalizerDE 1.13.2) was used to compare normalization strategies, with Variance Stabilizing Normalization selected for lowest intragroup variability.^43^

Features missing in >5% of patients were excluded to minimize imputation, acknowledging that this conservative approach may omit relevant signals. The remaining 0.25% of missing values were imputed using kNN (k = √478), yielding a final 478 × 3946 dataset (3300 peptides, 119 metabolites, and 527 lipids).

#### Differential expression analysis

Differential expression between PAH and CTL subjects was assessed using linear models equivalent to two-sample comparisons. False discovery rate was controlled using the Benjamini–Hochberg procedure. Features from each molecular layer were classified as upregulated or downregulated in PAH if |log2(fold change)| > 0.3 and adjusted *p* value <0.05 was satisfied. Counts and percentages of upregulated and downregulated features were summarized for each molecular layer.

#### Mixed graphical models

To evaluate potential interactions between predictive circulating molecules, patient demographics, hemodynamics, lung function, clinical bloodwork, and disease course (e.g., time from diagnosis), we trained a 2^nd^ order (pairwise comparisons) mixed graphical model using the mgm-package in R (L1-penalized regression, which is weighted by the extended Bayesian information criterion, γ = 0.25). The available PAH clinical data, not including the multi-omics data, had 4% missing values, which were imputed using kNN (k = $\sqrt{402}$).

#### Latent program discovery

We utilized significant latent factor interaction discovery and exploration (SLIDE^21^) to identify stand-alone and interacting latent networks that differentiate PAH from CTL subjects while revealing relationships across proteomic, metabolomic, and lipidomic layers. SLIDE captures both linear and nonlinear associations and provides statistical guarantees of latent factor identifiability.

Following hyperparameter optimization (optimal δ = 0.1 and λ = 0.5), performance of the latent factors was assessed using 10-fold cross-validation with 20 repeated resampling iterations.

In our dataset, 76 out of the 478 patients were CTLs. We chose not to apply special corrections for class imbalance for three reasons. First, our minority class represented about 16% of the data, which is considered a moderate imbalance. Second, the area under the ROC curve, the metric used in our SLIDE analysis, is known to be robust against moderate imbalance.^44^ Finally, the SLIDE results are meant to identify latent molecular programs rather than serve as direct biomarkers. Thus, our focus is on uncovering potential multi-omics programs that will be further scrutinized, not on classification performance alone.

#### Pairwise correlation models

Pairwise correlation networks were constructed to evaluate co-regulation among molecules within each latent factor in CTL and PAH subjects. Pearson correlations were computed using pairwise complete observations, and statistically meaningful edges were defined using a group-specific critical correlation corresponding to a two-sided α = 0.001 to account for differences in sample size between CTL and PAH cohorts. Node positions were determined using a force-directed layout and held fixed within each group for visualization consistency.

#### Statistical inference

For comparisons between two groups, two-tailed *t*-tests were used. Equality of variances was first assessed using an *F*-test; when variances were unequal, Welch’s two-sample *t* test was used. Comparisons among more than two groups were performed using analysis of variance (ANOVA), followed by Tukey–Kramer post hoc pairwise comparisons. Survival distributions were estimated using Kaplan–Meier analysis and compared using the log rank test. Statistical significance was defined as *p* < 0.05. Exact statistical tests, biological sample sizes, and *p* values for individual figure panels are provided in the figure legends or in Data S1 (STAR Methods).

## Supplemental information

Document S1. Figures S1–S3

Table S1. Peptide reference Data S1, demographics and statistical results

Method S1. Supplemental methods for multi-omics acquisition, SLIDE validation, and survival modeling
