# Supplementary material for: Identification of multi-omics molecular programs from the peripheral circulation of patients with PAH
Source: iScience. 2026 Nov 2;29(11):117937. doi: 10.1016/j.isci.2026.117937 (PMC13756107; doi:10.1016/j.isci.2026.117937)
Supplement: Supplementary file 1 [file mmc1.pdf]

iScience, Volume *29*

Supplemental information

Identification of multi-omics molecular programs from the peripheral circulation of patients with PAH

Vitaly O. Kheyfets, Andrew J. Sweatt, Hui Zhang, Min Li, Cheng-Jun Hu, Travis Nemkov, Maya A. Aizin, Paul Heerdt, Monika Dzieciatkowska, Daniel Stephenson, Ian S. LaCroix, Angelo D'Alessandro, Francesca I. Cendali, William M. Oldham, Kirk C. Hansen, Roham T. Zamanian, and Kurt R. Stenmark

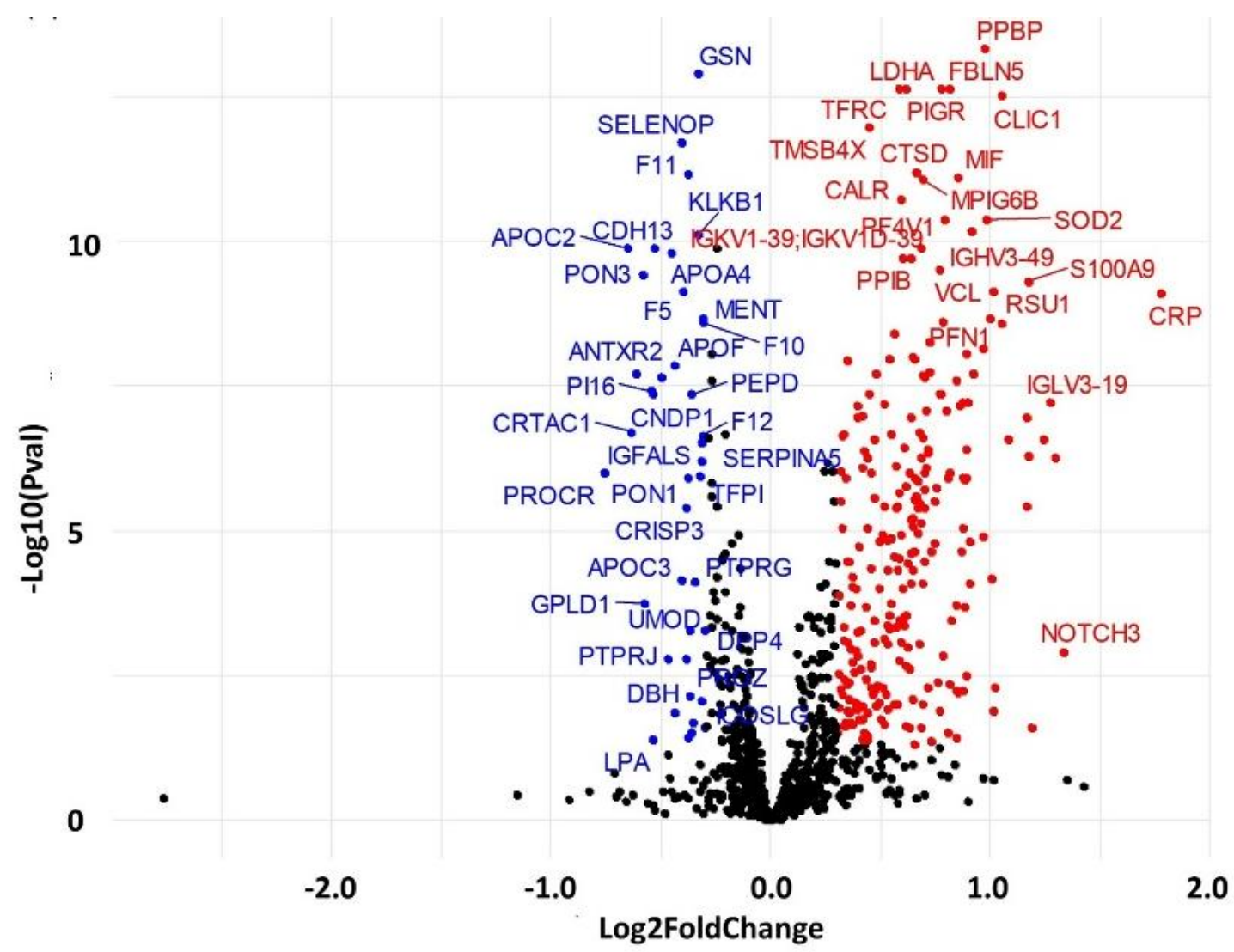

Figure S1. Volcano plot of circulating protein abundance. Red and blue markers indicate proteins up- and down-regulated in PAH, respectively.

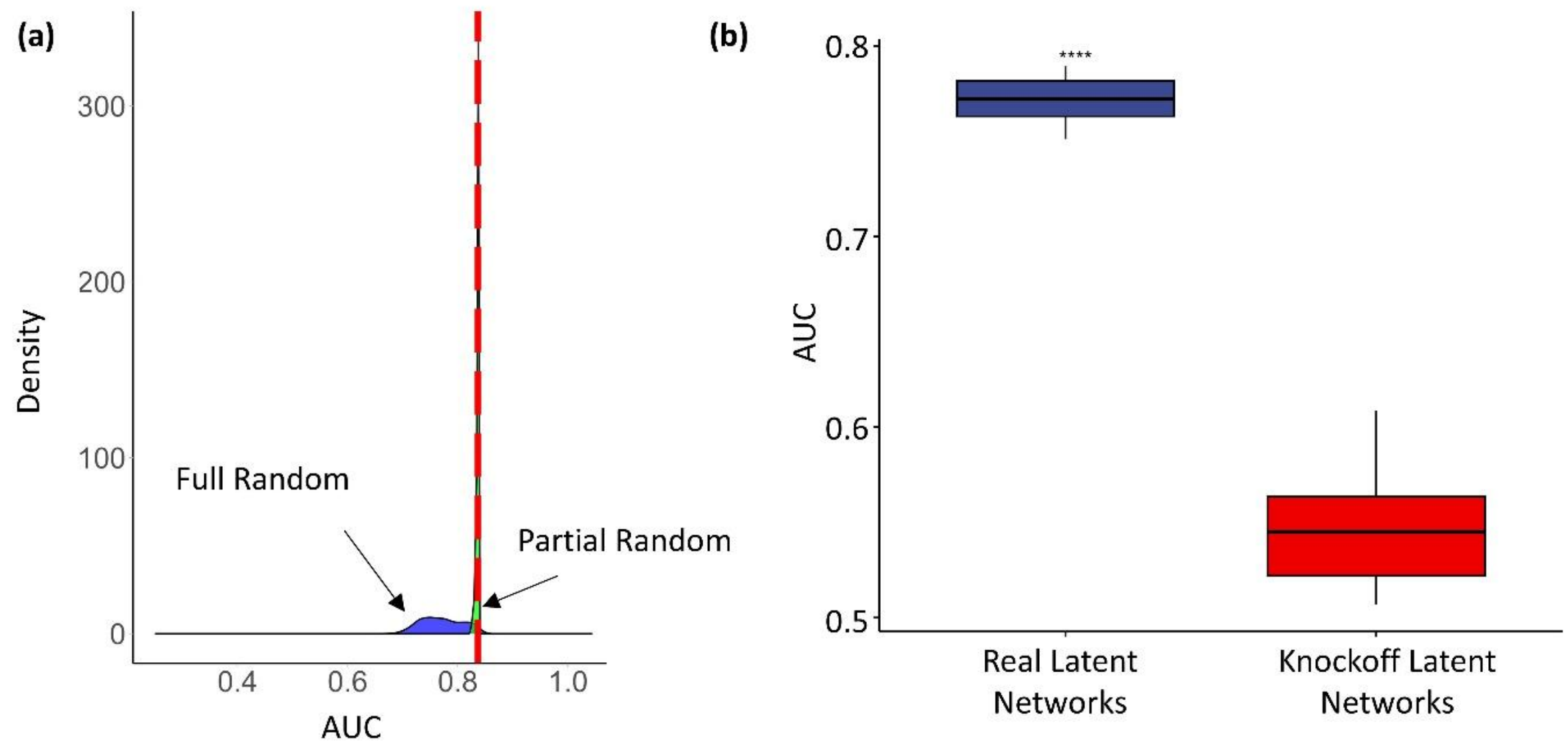

Figure S2. Validation of SLIDE model performance using knockoff controls. (a) SLIDE model performance. Red line shows actual performance of the significant underlying drivers. Blue density shows the performance of the "knockoffs" (a fake driver introduced into the algorithm), while green density shows the performance of the true marginal latent variables paired with knockoffs. AUC = area under curve. (b) Further evaluation of model performance.The blue boxplot shows the distribution of AUC values obtained from the true latent variables using 10-fold cross-validation with 20 repeated resampling iterations, demonstrating consistent prediction of PAH. The red boxplot shows AUC values derived from knockoff latent variables lacking biological signal. Real latent networks achieve significantly higher discriminative performance than knockoff networks (**** $p < 0.0001$), supporting that the identified latent structure captures biologically meaningful signal rather than noise.

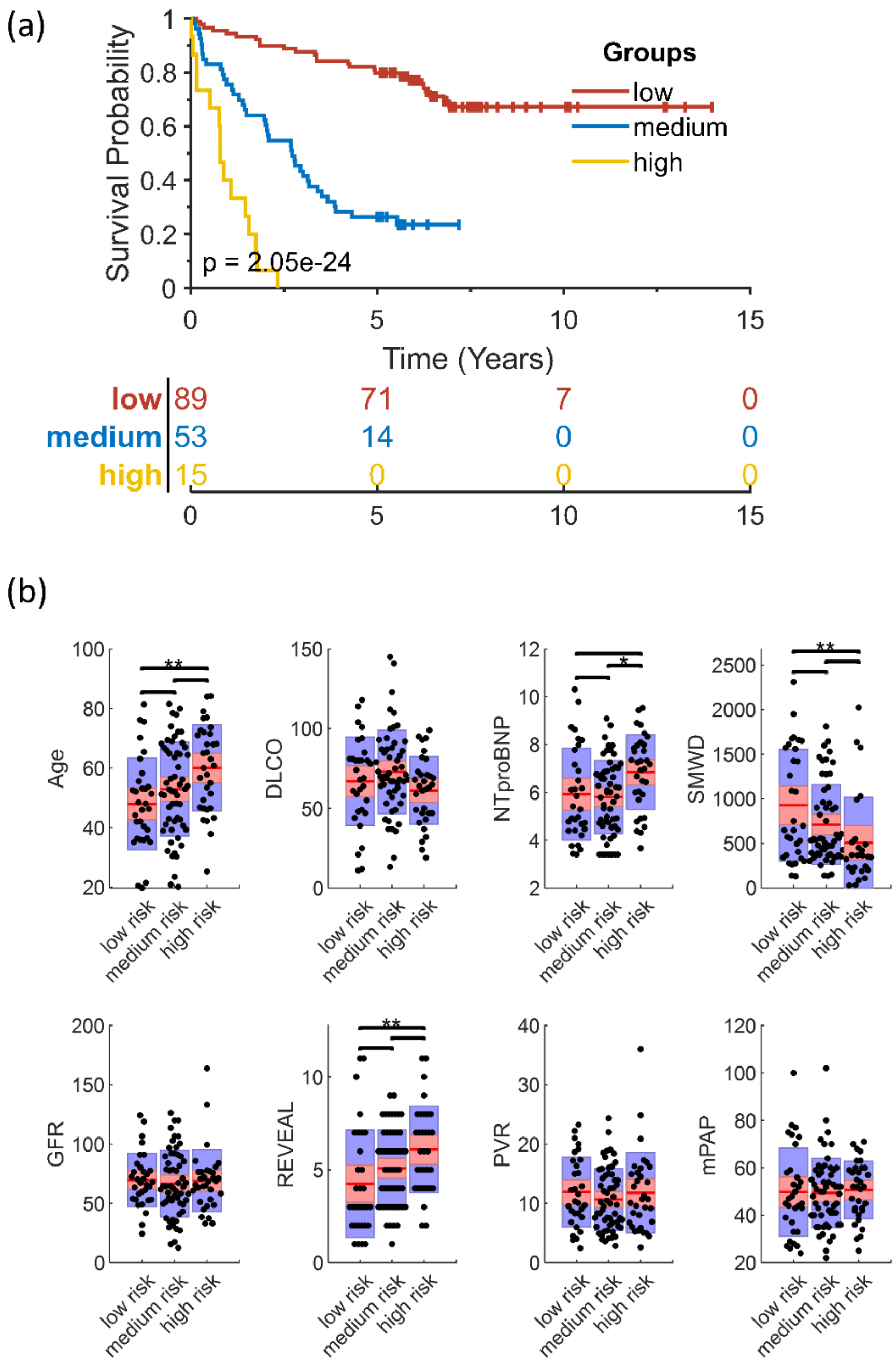

Figure S3. SLIDE latent networks stratify patients by survival risk. (a) Kaplan-Meier curves for patients in the testing cohort (N = 122) categorized by the RF survival model as low, medium, or high risk. Note: patients who were right-censored prior to the 5-year mark were not included in the Kaplan-Meier curves, which is why there are less than 122 subjects. (b) Demographic and clinical markers compared between patients categorized as low, medium, or high risk in the testing cohort (all 122 patients in the testing cohort considered). Note: mPAP: mean pulmonary arterial pressure; SMWD: six-minute walk distance. Reported p-values were computed by One-Way ANOVA with Tukey-Kramer for pairwise comparisons (*$p < 0.05$; **$p < 1e{-2}$; ***$p < 1e{-3}$).

Methods S1. Supplemental methods for multi-omics acquisition, SLIDE validation, and survival modeling

Mass Spectrometry and Multi-Omics Analysis for the primary cohort

Proteomics – Plasma samples were prepared as previously described[1]. Briefly, approximately 50ug plasma proteins were digested with trypsin using S-Trap 96-well plates (Protifi, Huntington, NY, USA) following the manufacturer procedures. Peptides were separated using an Evosep One system using the 30 runs per day method (Evosep, Odense, Denmark) and a Pepsep column. The system was coupled to a timsTOF MS Pro (Bruker Daltonics, Bremen Germany) via electro spray ionization. The MS was operated in diaPASEF mode. Raw DIA files were searched using Spectronaut v 17.0 (Biognosys, Schlieren, Switzerland), using a project-specific spectral library from DDA runs of 24 fractions and searched with the Pulsar search engine.

Fig. S1 shows a volcano plot comparing protein abundance between control and PAH patients.

Metabolomics and Lipidomics –High throughput metabolomics and lipidomics were performed via ultra-high performance liquid chromatography coupled to high-resolution mass spectrometry, consistent with previously published methods[2]. A volume of 480 uL of ice cold 5:3:2 MeOH:MeCN:water (v/v/v) or 180 uL pure methanol was added to 20 uL of each sample for metabolomics and lipidomics, respectively, prior to vortexing for 30 min at 4ºC and centrifugation for 10 min at 18,000*g* at 4ºC. Blanks and quality control samples (generated by mixing 5 uL of all sample extracts) were injected every 20 samples and used to monitor instrument performance throughout the analysis. Metabolites were resolved on a Phenomenex Kinetex C18 column (2.1 x 30 mm, 1.7 um) at 45 °C using a 1-minute ballistic gradient method in positive and negative ion modes (separate runs) over the scan range 65-975 m/z exactly as previously described[3]. The UHPLC was coupled online to a Q Exactive mass spectrometer (Thermo Fisher). The Q Exactive MS was operated in negative ion mode, scanning in Full MS mode (2 μscans) from 90 to 900 m/z at 70,000 resolution, with 4 kV spray voltage, 45 sheath gas, 15 auxiliary gas. Following data acquisition, .raw files were converted to .mzXML using RawConverter then metabolites assigned and peaks integrated using ElMaven (Elucidata) in conjunction with an in-house standard library[4].

Untargeted lipidomics analyses were performed via UHPLC-MS/MS, as previously described[5]. Briefly, lipidomics analysis employed a Vanquish UHPLC system (Thermo Fisher Scientific) coupled to a Q Exactive mass spectrometer (Thermo Fisher Scientific). A volume of 5 μL injections of the samples were resolved across a 2.1 x 30 mm, 1.7 um particle size Kinetex C18 column (Phenomenex) using a 5 minute, reversed-phase gradient adapted from a previous method. The Q Exactive was run independently in positive and negative ion mode, scanning using data dependent MS^2 (top 10) from 125-1500 m/z at 17,500 resolution, with 45 Arb sheath gas, 25 Arb auxiliary gas, and 4 kV spray voltage. Calibration was performed prior to the run using the PierceTM Positive and Negative Ion Calibration Solutions (Thermo Fisher Scientific). Run order of samples was randomized and technical replicates were injected after every 4

samples to assess quality control. Lipid assignments and peak integration were performed using LipidSearch v 5.0 (Thermo Fisher Scientific).

Cross-Validation and Robustness of Latent Factors

To reduce the dimensionality of the integrated proteomic, metabolomic, and lipidomic dataset and uncover coordinated molecular programs associated with pulmonary arterial hypertension (PAH), we applied the SLIDE framework to identify stand-alone and interacting latent networks that differentiate PAH from control (CTL) subjects. This approach is grounded in the assumption that high-dimensional multi-omics data arise from a lower-dimensional latent structure. Specifically, the measured circulating features are assumed to be generated from a smaller set of latent variables, each representing a weighted linear combination of subsets of proteins, metabolites, and lipids. In this manuscript, these lower-dimensional representations are referred to as *latent factors (sometimes latent networks)*.

Conceptually, each latent network captures a co-regulated molecular program spanning multiple omics layers. By examining the composition and interactions of these networks, we gain insight into which circulating molecules move together and how proteomic, metabolomic, and lipidomic features coordinate to distinguish PAH from CTL subjects. Importantly, SLIDE incorporates both linear and nonlinear relationships among features and provides statistical guarantees regarding latent factor identifiability[6], ensuring that the derived latent structure is uniquely and stably defined under model assumptions.

Hyperparameter optimization was performed in R (version 4.2.2), yielding an optimal configuration of $\delta = 0.1$ and $\lambda = 0.5$. Candidate models were evaluated using repeated cross-validation. For the selected model, performance of the significant latent networks was assessed using 10-fold cross-validation with 20 repeated resampling iterations to evaluate generalizability and guard against overfitting.

Figure S2a presents the overall model performance. The red dashed vertical line denotes the observed area under the receiver operating characteristic curve (AUC) achieved by the significant latent networks derived from the true data. The blue density represents fully randomized latent factors used for false discovery control, while the green density corresponds to knockoff latent variables paired with significant networks. In both cases, performance is lower than that of the true latent structure, indicating that the discriminative ability of the model is not attributable to random feature combinations or correlation structure alone.

Figure S2b further evaluates model robustness through repeated cross-validation. The blue boxplot displays the distribution of AUC values obtained from the true latent variables across 10-fold cross-validation with 20 resampling iterations, demonstrating strong and consistent prediction of PAH. In contrast, the red boxplot shows AUC values derived from knockoff latent variables, which are artificial variables constructed to preserve data structure while removing biological signal. These knockoff variables yield substantially lower AUC values, showing almost random classification performance.

Together, these analyses demonstrate that the identified latent networks represent stable, disease-associated molecular programs. Their predictive performance generalizes across resampling and is significantly stronger than that obtained from randomized or knockoff structures, supporting the conclusion that SLIDE is capturing biologically meaningful patterns rather than overfitting to noise.

Association between latent variables and disease severity

Using the circulating biomarkers identified within the SLIDE-derived latent space (the compounds with the highest feature loadings and the strongest univariate AUC for discriminating PAH from CTL subjects) we trained a random survival forest (1000 trees, a minimum of 15 samples required to split an internal node, a minimum of 15 samples required to be in a leaf node) on a training subset of 280 PAH patients from the primary cohort, with 122 patients withheld for testing.

Assessing the model's performance on the reserved testing subset yielded a concordance index of 0.74. Patients were divided into three groups based on their percentile of the predicted 5-year cumulative hazard: (1) Low risk (≤33rd percentile); (2) Medium risk (33rd to 67th percentile); and (3) High risk (>67th percentile). Patients in the testing subset were stratified based on percentile thresholds identified in the training subset to avoid biasing results. Fig. S3a shows Kaplan-Meier survival curves for the three risk groups in the testing cohort. Patients who did not experience an event but were last evaluated less than 5 years after their blood draw (right-censored prior to 5 years) were excluded from these curves, which is why fewer than 122 subjects appear.

When comparing model prediction to clinical data, the RSF model predictions were consistent with known clinical risk factors. As shown in Fig. S3b, patients in the testing cohort assigned to the high-risk group were older, had elevated NT-proBNP levels, reduced exercise capacity (SMWD), and a higher REVEAL 2.0 risk score compared to those in the low-risk group.

Supplement References

1. LaCroix, I.S., Cohen, M., Moore, E.E., Dzieciatkowska, M., Nemkov, T., Schaid, T.R., Jr., Debot, M., Jones, K., Silliman, C.C., Hansen, K.C., and D'Alessandro, A. (2022). Omics Markers of Red Blood Cell Transfusion in Trauma. Int J Mol Sci *23*. 10.3390/ijms232213815.
2. D'Alessandro, A. High-Throughput Metabolomics: Methods and Protocols (Springer Nature).
3. Nemkov, T., Yoshida, T., Nikulina, M., and D'Alessandro, A. (2022). High-Throughput Metabolomics Platform for the Rapid Data-Driven Development of Novel Additive Solutions for Blood Storage. Front Physiol *13*, 833242. 10.3389/fphys.2022.833242.
4. Nemkov, T., Hansen, K.C., and D'Alessandro, A. (2017). A three-minute method for high-throughput quantitative metabolomics and quantitative tracing experiments of central carbon and nitrogen pathways. Rapid Commun Mass Spectrom *31*, 663–673. 10.1002/rcm.7834.
5. Reisz, J.A., Zheng, C., D'Alessandro, A., and Nemkov, T. (2019). Untargeted and Semi-targeted Lipid Analysis of Biological Samples Using Mass Spectrometry-Based Metabolomics. Methods Mol Biol *1978*, 121–135. 10.1007/978-1-4939-9236-2_8.
6. Rahimikollu, J., Xiao, H., Rosengart, A., Rosen, A.B.I., Tabib, T., Zdinak, P.M., He, K., Bing, X., Bunea, F., Wegkamp, M., et al. (2024). SLIDE: Significant Latent Factor Interaction Discovery and Exploration across biological domains. Nature Methods *21*, 835–845. 10.1038/s41592-024-02175-z.
